# Whole blood‐based measurement of SARS‐CoV‐2‐specific T cells reveals asymptomatic infection and vaccine immunogenicity in healthy subjects and patients with solid‐organ cancers

**DOI:** 10.1111/imm.13433

**Published:** 2021-12-06

**Authors:** Martin J. Scurr, Wioleta M. Zelek, George Lippiatt, Michelle Somerville, Stephanie E. A. Burnell, Lorenzo Capitani, Kate Davies, Helen Lawton, Thomas Tozer, Tara Rees, Kerry Roberts, Mererid Evans, Amanda Jackson, Charlotte Young, Lucy Fairclough, Paddy Tighe, Mark Wills, Andrew D. Westwell, B. Paul Morgan, Awen Gallimore, Andrew Godkin

**Affiliations:** ^1^ Division of Infection & Immunity School of Medicine Cardiff University Cardiff UK; ^2^ ImmunoServ Ltd Cardiff UK; ^3^ School of Medicine Systems Immunity University Research Institute Cardiff University Cardiff UK; ^4^ UK Dementia Research Institute Cardiff Cardiff University Cardiff UK; ^5^ Radyr Medical Centre Cardiff UK; ^6^ Department of Gastroenterology & Hepatology University Hospital of Wales Cardiff UK; ^7^ Velindre Cancer Centre Cardiff UK; ^8^ School of Life Sciences University of Nottingham Nottingham UK; ^9^ Department of Medicine Addenbrooke’s Hospital University of Cambridge Cambridge UK; ^10^ School of Pharmacy and Pharmaceutical Sciences Cardiff University Cardiff UK

**Keywords:** antibodies, COVID‐19, SARS‐CoV‐2, T cells, vaccine

## Abstract

Accurate assessment of SARS‐CoV‐2 immunity is critical in evaluating vaccine efficacy and devising public health policies. Whilst the exact nature of effective immunity remains incompletely defined, SARS‐CoV‐2‐specific T‐cell responses are a critical feature that will likely form a key correlate of protection against COVID‐19. Here, we developed and optimized a high‐throughput whole blood‐based assay to determine the T‐cell response associated with prior SARS‐CoV‐2 infection and/or vaccination amongst 231 healthy donors and 68 cancer patients. Following overnight *in vitro* stimulation with SARS‐CoV‐2‐specific peptides, blood plasma samples were analysed for T_H_1‐type cytokines. Highly significant differential IFN‐γ^+^/IL‐2^+^ SARS‐CoV‐2‐specific T‐cell responses were seen amongst previously infected COVID‐19‐positive healthy donors in comparison with unknown / naïve individuals (*p* < 0·0001). IFN‐γ production was more effective at identifying asymptomatic donors, demonstrating higher sensitivity (96·0% vs. 83·3%) but lower specificity (84·4% vs. 92·5%) than measurement of IL‐2. A single COVID‐19 vaccine dose induced IFN‐γ and/or IL‐2 SARS‐CoV‐2‐specific T‐cell responses in 116 of 128 (90·6%) healthy donors, reducing significantly to 27 of 56 (48·2%) when measured in cancer patients (*p* < 0·0001). A second dose was sufficient to boost T‐cell responses in the majority (90·6%) of cancer patients, albeit IFN‐γ^+^ responses were still significantly lower overall than those induced in healthy donors (*p* = 0·034). Three‐month post‐vaccination T‐cell responses also declined at a faster rate in cancer patients. Overall, this cost‐effective standardizable test ensures accurate and comparable assessments of SARS‐CoV‐2‐specific T‐cell responses amenable to widespread population immunity testing, and identifies individuals at greater need of booster vaccinations.

## Graphical abstract

SARS‐CoV‐2 pandemic continues to cause morbidity and mortality especially in vulnerable patients. Monitoring individuals T and B cell responses to the virus is desirable in high risk populations after infection and/or vaccination. This paper describes a straightforward approach on a small whole blood sample to measure these responses.

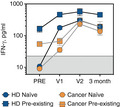

**Abbreviations**
COVID‐19: coronavirus disease 2019
ELISpot: enzyme‐linked immunospot
IFN‐γ: Interferon‐gamma
IgG: immunoglobulin G
IL‐2: interleukin‐2
PHA: phytohaemagglutinin
SARS‐CoV‐2: severe acute respiratory syndrome, coronavirus 2
T_H_1: T‐helper type 1

## BACKGROUND

As the COVID‐19 pandemic continues, there is an increasing focus on understanding how adaptive immune responses generated from severe acute respiratory syndrome coronavirus 2 (SARS‐CoV‐2) infection and/or vaccination provide protection from future infection. Although the exact determinants of effective immunity from reinfection with SARS‐CoV‐2 remain to be deciphered, multiple recent studies have revealed that virus‐specific T‐cell responses develop in nearly all individuals with confirmed SARS‐CoV‐2 infection [1, 2, 3, 4], with responses persisting for at least six months post‐infection [5]. Traditional means of assessing viral antigen‐specific T‐cell responses utilize flow cytometry or ELISpot‐based read‐outs; however, neither approach is standardizable across multiple laboratories, cost‐effective or amenable to high‐throughput processing, thus precluding their use for larger scale population immunity screens. In addition, current commercially available immunoassays that detect cellular immune responses to SARS‐CoV‐2 solely measure IFN‐γ released by antigen‐specific T cells [6], even though other T_H_1‐type cytokines may be better indicators of antiviral response [5]. To overcome these limitations, existing whole blood‐based *in vitro* immunodiagnostics, such as those used for measuring Mycobacterium tuberculosis‐specific T‐cell responses [7], can be adapted to measure virus‐specific T‐cell responses in a high‐throughput, standardizable manner. Specifically, this T‐cell immunoassay measures cytokines in the plasma released by antigen‐specific T cells following stimulation with specific peptides spanning antigenic regions of the pathogen. This approach is gaining recognition as a potentially powerful diagnostic tool for managing the COVID‐19 pandemic [8, 9, 10].

Monitoring immunological responses to SARS‐CoV‐2 is of particular importance amongst the elderly and immunosuppressed, given the significantly higher mortality rates observed in these groups [11]. Recent studies have associated higher rates of COVID‐19 morbidity and mortality with suboptimal adaptive immune responses [12]. The incidence of cancer is also increased in the elderly where a declining adaptive immune system and age‐associated inflammation are factors in disease progression. Given that influenza vaccines induce weaker immune responses in the elderly and in cancer patients [13, 14], measuring adaptive immune responses to SARS‐CoV‐2 in vaccinated individuals belonging to these groups is important. Indeed, early indications suggest that cancer patients, in particular those on active treatments such as chemotherapy, were significantly less likely to mount antibody and T‐cell responses to the Pfizer‐BioNTech SARS‐CoV‐2 mRNA vaccine, [15].

Here, we adapted and optimized a widely utilized *in vitro* whole‐blood stimulation assay to determine the presence of SARS‐CoV‐2‐specific T_H_1‐type (IFN‐γ/IL‐2) cellular immune responses in healthy donors, to assess T‐cell responses generated from prior infection, whether the participant was symptomatic or not, and as a read‐out of vaccine immunogenicity amongst healthy donors and cancer patients. We demonstrate high sensitivity and specificity of this assay to identify or exclude prior SARS‐CoV‐2 infection and/or successful COVID‐19 vaccination. Going forward, it is imperative to utilize such tests to understand the precise contribution of T‐cell responses with regard to long‐term immunity to SARS‐CoV‐2 infection, in particular amongst immunocompromised individuals.

## METHODS

### Study cohorts

Participants were recruited to this research project between February and April 2021. A healthy donor cohort (*n* = 231) comprised university staff and students attending Cardiff University's COVID‐19 Screening Service or members of the public attending a Cardiff‐based GP practice. All participants were otherwise healthy and did not report taking any current immunosuppressive medication. In addition, patients with a range of solid‐organ cancers (*n* = 68) were recruited from Velindre Cancer Centre prior to receiving their first COVID‐19 vaccine (see Table 1 for patient characteristics). All participants were stratified based on self‐reported and/or laboratory evidence of a prior SARS‐CoV‐2 infection. Participants reporting no prior positive test were defined as ‘unknown/naïve’. To measure immunological responses generated to COVID‐19 vaccination, baseline blood samples were taken immediately preceding the first dose; additional post‐vaccination blood samples were taken 3–6 weeks following each dose and at least 3 months following the second dose. All vaccinated participants received either Pfizer (BNT162b2) mRNA vaccine or AstraZeneca (ChAdOx1 nCoV‐19) vaccine (Table 1).

**TABLE 1:** Participant characteristics

	Cancer patients (*n* = 68)	Healthy donors (*n* = 231)
Mean age, years (range)	52·6 (28–79)	41·4 (18–81)
Sex		
Male	25/68 (36·8%)	81/231 (35·1%)
Female	43/68 (63·3%)	150/231 (64·9%)
Vaccine status		
Pfizer (BNT162b2)	68/68 (100·0%)	95/231 (41·1%)
AstraZeneca (ChAdOx1 nCoV−19)	0/68 (0%)	76/231 (32·9%)
Unknown vaccine	0/68 (0%)	2/231 (0·9%)
Unvaccinated^a^	0/68 (0%)	58/231 (25·1%)
Malignancy		
Breast	20/68 (29·4%)	N/A
Gastrointestinal	16/68 (23·5%)	N/A
Prostate	8/68 (11·8%)	N/A
Lung	5/68 (7·4%)	N/A
Female reproductive	5/68 (7·4%)	N/A
Melanoma	3/68 (4·4%)	N/A
Brain	3/68 (4·4%)	N/A
Other	8/68 (11·8%)	N/A
Cancer treatment on study		
Chemotherapy	22/68 (32·4%)	N/A
Immunotherapy	7/68 (10·3%)	N/A
Radiotherapy	3/68 (4·4%)	N/A
Hormone treatment	14/68 (20·6%)	N/A
Tyrosine kinase inhibitors	11/68 (16·2%)	N/A
Not on treatment	11/68 (16·2%)	N/A

^a^ Indicates participants where only a pre‐vaccination blood sample was obtained.

This study received ethical approval from the Wales Cancer Bank (WCB No. 21/004), the Newcastle & North Tyneside 2 Research Ethics Committee (IRAS ID: 294246) and Cardiff University School of Medicine Research Ethics Committee (SREC reference: SMREC 21/01). All participants gave written, informed consent prior to inclusion.

### Peptides

All SARS‐CoV‐2 peptides were dissolved according to the manufacturer's instructions (Miltenyi Biotec). A single SARS‐CoV‐2 (wild‐type variant)‐specific peptide pool was created, comprising 420 15‐mer sequences with 11 amino acid overlap, covering the entire spike (S1 and S2) protein (S; NCBI Protein: QHD43416·1), nucleocapsid phosphoprotein (NP; NCBI Protein: QHD43423·2) and membrane glycoprotein (M; NCBI Protein: QHD43419·1) coding sequences (termed ‘S‐/NP‐/M‐combined peptide pool’). All peptides were purified to >70%, dissolved in sterile water and used at a final concentration of 0·5μg/ml per peptide.

### Stimulation

A single 6‐ml or 10‐ml sodium heparin vacutainer (BD) tube of venous blood was collected from each participant and processed in the laboratory within 12 h of blood draw. 1ml whole‐blood samples were aliquoted into microcentrifuge tubes (Thermo Scientific) containing 30 μl S‐/NP‐/M‐combined peptide pool, alongside additional tubes containing 20 μg/ml phytohaemagglutinin (Sigma) (positive control) or nothing (negative control). Samples were incubated at 37°C for 20–24 h. Tubes were then centrifuged at 3000 g for 2 min before harvesting ~150 μl plasma from the top of each blood sample. Plasma samples were stored at −20°C for up to one month prior to running cytokine detection assays.

### Detection of anti‐SARS‐CoV‐2 RBD IgG Antibodies

An in‐house direct ELISA was developed as previously described [16, 17, 18, 19], with some modifications. MaxiSorp (Nunc, Loughborough, UK) 96‐well plates were coated with RBD protein (recombinantly generated in a mammalian expression system, in‐house) at 2 μg/ml in bicarbonate buffer, pH 9·6 at 4°C overnight; wells were blocked for 1 h at room temperature with 3% w/v non‐fat dried milk powder (Sigma‐Aldrich, # 70166‐500G) in phosphate‐buffered saline containing 0·1% Tween‐20 (PBS‐T), washed in PBS‐T. Dilutions of patient sera (1 in 50 in 1% milk PBS‐T) were added in duplicate to wells coated with RBD protein and incubated for 2 h at room temperature. Wells were washed three times with PBS‐T, then incubated (1 h, room temperature) with secondary antibody (donkey anti‐human IgG F(ab’)₂‐horseradish peroxidase (HRP); #709‐036–149, Jackson ImmunoResearch, Ely, UK) for 1 h at room temperature. After washing (x3), plates were developed using O‐phenylenediamine dihydrochloride (OPD, SIGMAFASTTM; Sigma‐Aldrich, # P9187‐50SET), and the optical density (OD) was measured at 492 nm. Assay validation, including intra‐/inter‐assay CVs for this assay, has been previously described [17, 19].

### Detection of anti‐SARS‐CoV‐2 S1 / S2 / N IgG antibodies

Anti‐SARS‐CoV‐2 S1/S2/N IgG antibodies were measured using the Bio‐Plex Pro Human SARS‐CoV‐2 N/RBD/S1/S2 4‐plex panel (Bio‐Rad) and performed according to the manufacturer's instructions. The mean fluorescent intensity of the beads was measured on a Bio‐Plex 200 (Bio‐Rad). Antibody concentration was calculated by performing the assay with the VIROTROL SARS‐CoV‐2 single‐level control (Bio‐Rad).

### Cytokine Detection

IFN‐γ was measured using the IFN‐γ ELISA MAX Deluxe Kit (BioLegend) and performed according to the manufacturer's instructions. Microplates were read at 450nm immediately following the addition of stop solution (2N H_2_SO_4_). IFN‐γ was quantified by extrapolating from the standard curve using GraphPad Prism. Values below the lower limit of detection of the assay were recorded as 7·81 pg/ml. IL‐2 was measured using a custom Bio‐Plex Pro Human Cytokine Set (Bio‐Rad) and performed according to the manufacturer's instructions. The mean fluorescent intensity of the cytokine beads was measured on a Bio‐Plex 200 (Bio‐Rad). Cytokine concentration was calculated from control curves of standards provided in the kit. Values below the lower limit of detection of the assay were recorded as 6·28 pg/ml.

### Statistics

GraphPad Prism version 9 was used for all statistical analyses of data sets. Data set normality was tested using the Shapiro–Wilk test. Significance was determined using either Fisher's exact tests, Kruskal–Wallis tests with corrections for multiple comparisons made using the Dunn test, Wilcoxon matched‐pairs signed rank tests or Mann‐Whitney tests, as indicated in the figure legends. Correlation analyses were performed using the linear regression analysis. All tests were performed two‐sided with a nominal significance threshold of *p* < 0·05. Statistical significance was either stated numerically, or abbreviated on some figures using asterisk symbols as follows: *indicates *p* < 0·05; ** indicates *p* < 0·01 *** indicates *p* < 0·001**** indicates *p* < 0·0001.

## RESULTS

**FIGURE 1:**
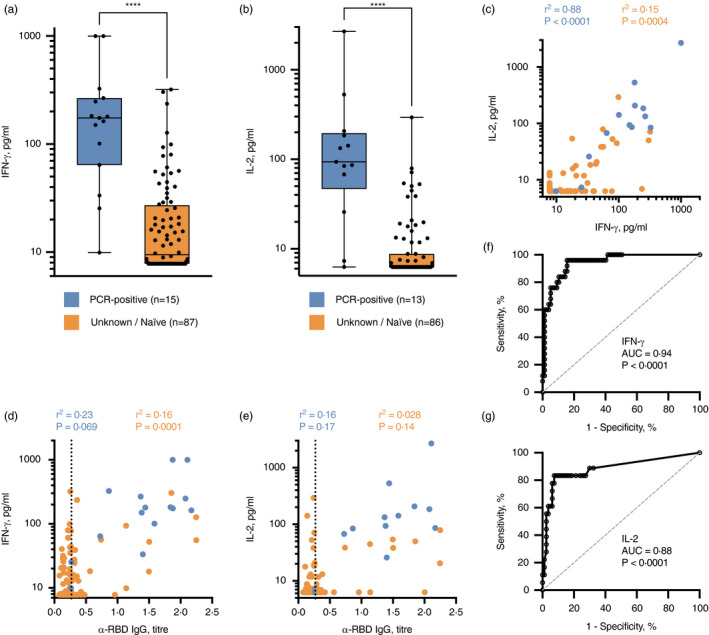
SARS‐CoV‐2‐specific T‐cell response identifies previously infected individuals. IFN‐γ (A) and IL‐2 (B) release in response to the SARS‐CoV‐2 S‐/NP‐/M‐combined peptide pool was measured in 102 evaluable, non‐vaccinated participants, subdivided into those with prior COVID‐19‐positive PCR test result (blue, *n* = 13–15), or those with no prior positive COVID‐19 test, termed ‘unknown/naïve’ (orange, *n* = 86–87). Statistical analyses indicate the results of a Kruskal–Wallis test (**** *p* < 0·0001). (C) The SARS‐CoV‐2‐specific IL‐2^+^ T‐cell response was correlated with the IFN‐γ^+^ response, subdivided by participant status. The anti‐SARS‐CoV‐2 RBD IgG antibody titre was correlated with the magnitude of IFN‐γ^+^ T‐cell response (D) and IL‐2^+^ T‐cell response (E); results of regression analyses are indicated. Sensitivity and specificity read‐outs for IFN‐γ (F) and IL‐2 (G) were defined by receiver operating characteristic curves (optimal cut‐off for IFN‐γ 22·7 pg/ml; IL‐2 23·31 pg/ml). The area under the curve (AUC) and associated P value are indicated

Prior analysis of the SARS‐CoV‐2‐specific T‐cell cytokine profile, measured in supernatants of *ex vivo* peptide‐stimulated ELISpot cultures or whole‐blood assays, revealed that T_H_1‐type IFN‐γ^+^ and IL‐2^+^ responses dominate effective, functional SARS‐CoV‐2‐specific T‐cell responses [5, 10]. We utilized a whole‐blood stimulation assay to assess the magnitude of T_H_1‐type responses generated to the SARS‐CoV‐2 S‐/NP‐/M‐combined peptide pool in a high‐throughput format (Figure S1). T‐cell responses were measured in healthy donor non‐vaccinated participants who had a confirmed prior infection (*n* = 15) (PCR‐positive swab from nasopharynx or saliva sample and/or a strong history of infection with positive measured antibody responses to SARS‐CoV‐2) or no known history of infection (*n* = 87). Highly significant differential IFN‐γ‐positive and IL‐2‐positive SARS‐CoV‐2‐specific T‐cell responses were seen amongst previously infected COVID‐19‐positive individuals in comparison with unknown/naïve individuals (Figure 1A; *p* < 0·0001 and Figure 1B; *p* < 0·0001, respectively). Amongst all infected or unknown/naïve participants, there were highly significant correlations between IFN‐γ and IL‐2 production, despite a propensity for increased (>20 pg/ml) IFN‐γ^+^ SARS‐CoV‐2‐specific T‐cell responses without a measurable IL‐2 response amongst unknown/naïve donors (Figure 1C).

Next, we correlated participant T‐cell responses with the presence of anti‐SARS‐CoV‐2 IgG antibodies. There was a strong concordance, albeit not significant correlation, between the presence of IFN‐γ^+^ (Figure 1D) or IL‐2^+^ (Figure 1E) responses and anti‐SARS‐CoV‐2 RBD IgG immune responses in previously infected participants: only 1/15 (6·7%) previously infected participant had a positive antibody response (>0·27) without a measurable IFN‐γ^+^ or IL‐2^+^ T‐cell response. A significant correlation between IFN‐γ^+^ T‐cell response and the magnitude of anti‐SARS‐CoV‐2 RBD IgG antibodies was noted for unknown/naïve participants (*r* ^2^ = 0·16, *p* = 0·0001, Figure 1D); the correlation between IL‐2^+^ T‐cell response and magnitude of RBD IgG was not significant (*r* ^2^ = 0·028, *p* = 0·14; Figure 1E). It is clear from these data that there are participants amongst the unknown / naïve cohort that exhibit adaptive immune responses consistent with those generated in the majority of previously infected participants.

When assessing the utility of measuring SARS‐CoV‐2‐specific T‐cell responses to identify those with confirmed prior infection, Youden's index revealed an optimal cut‐off value of >22·70 pg/ml IFN‐γ, achieving a sensitivity of 96·0% (95% CI 80·5–99·8%) and specificity of 84·4% (95% CI 74·7–90·9%) (AUC = 0·94 (95% CI 0·90–0·99); *p* < 0·0001; Figure 1F). Measuring IL‐2^+^ T‐cell response as the read‐out adjusted the sensitivity and specificity to 83·3% (95% CI 60·8–94·2%) and 92·5% (95% CI 84·6–96·5%), respectively, at an optimal cut‐off value of >23·31 pg/ml IL‐2 (AUC = 0·88 (95% CI 0·78–0·99); *p* < 0·0001; Figure 1G).

To investigate whether the increased T‐cell responses observed amongst naïve/unknown participants with no history of confirmed infection were indicative of asymptomatic SARS‐CoV‐2 infection, we further compared the magnitude of IFN‐γ^+^ or IL‐2^+^ T‐cell responses with evidence of antibody seroconversion; eleven such participants were identified as having both a positive anti‐RBD IgG response (>0·27) and positive IFN‐γ^+^ and/or IL‐2^+^ T‐cell response using the above criteria for defining positive/negative cut‐offs. Raised levels of anti‐spike 1, anti‐spike 2 or anti‐nucleocapsid phosphoprotein IgG were also noted (Figure S2). Given these participants self‐reported no prior confirmed COVID‐19 test or symptoms associated with COVID‐19 during the pandemic, the presence of both a SARS‐CoV‐2‐specific T‐cell and antibody response is highly indicative of prior asymptomatic infection.

**FIGURE 2:**
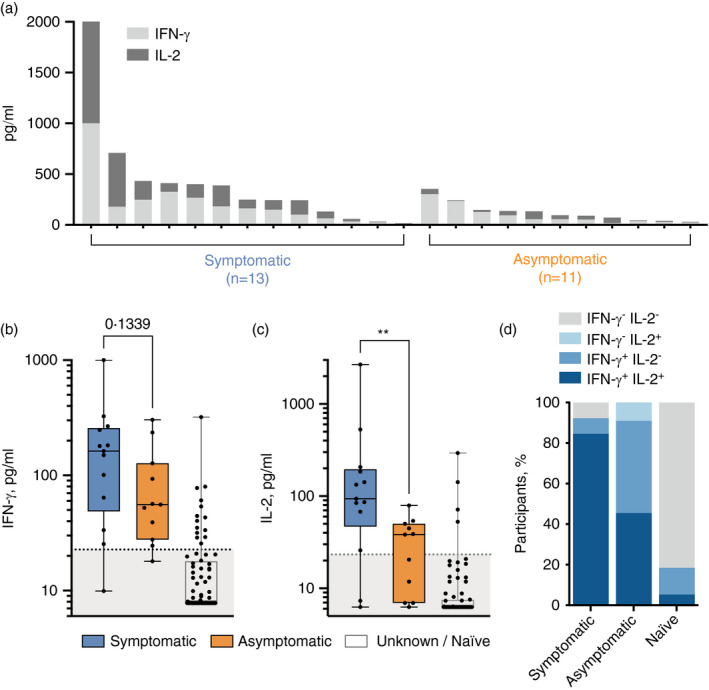
Asymptomatic participants exhibit reduced SARS‐CoV‐2‐specific T‐cell functionality. (A) IFN‐γ and IL‐2 T‐cell responses amongst thirteen symptomatic and eleven asymptomatic SARS‐CoV‐2‐infected donors are shown for each individual. IFN‐γ (B) and IL‐2 (C) release by T cells in response to the SARS‐CoV‐2 S‐/NP‐/M‐combined peptide pool was measured in the symptomatic, asymptomatic and naïve donors (*n* = 76). P values resulting from Mann–Whitney tests are shown (**p < 0·01). The proportion of symptomatic, asymptomatic and naïve participants mounting dual IFN‐γ^+^/IL‐2^+^, single IFN‐γ or IL‐2^+^ or no measurable T‐cell response is shown (D)

To determine the functionality of SARS‐CoV‐2‐specific T cells induced by prior asymptomatic infection, we further evaluated IFN‐γ and IL‐2 responses in these eleven asymptomatic, non‐vaccinated participants reporting no prior associated symptoms against thirteen non‐vaccinated COVID‐19‐positive convalescent participants reporting mild‐to‐moderate severity of associated symptoms (Figure 2A). The magnitude of IFN‐γ production was reduced in asymptomatic participants (*p* = 0·13, Figure 2B); furthermore, a significant reduction in IL‐2 production was noted (*p* = 0·0062, Figure 2C). When assessing all T_H_1 responses by participant infection and symptom status and using optimal cut‐offs defined above to identify positive responses, dual‐producing IFN‐γ^+^/IL‐2^+^ SARS‐CoV‐2‐specific T‐cell responses were present in 11 of 13 (84·6%) symptomatic participants, reducing to 5 of 11 (45·5%) amongst asymptomatic participants (Figure 2D). Given the diminished nature of IL‐2 production in SARS‐CoV‐2‐specific T_H_1‐type cells amongst asymptomatic donors, IFN‐γ is a more reliable read‐out when assessing for the presence of T‐cell responsiveness in participants with unknown infection history or vaccination status.

SARS‐CoV‐2‐specific T‐cell responses amongst unknown / naïve participants, that is those with no measurable anti‐SARS‐CoV‐2 IgG antibodies and no prior confirmed history of infection, were rare, with 4 of 76 (5·3%) displaying a dual IFN‐γ^+^/IL‐2^+^ response and 10 of 76 (13·2%) displaying an IFN‐γ^+^/IL‐2^−^ response (Figure 2D). This could be indicative of pre‐existing, cross‐reactive T cells, or a terminally differentiated effector T‐cell response [20]. However, further studies are required to ascertain whether those participants with raised anti‐SARS‐CoV‐2‐specific T_H_1 responses without seroconversion have been infected by SARS‐CoV‐2. Interestingly, this discordant immune response has also been reported when monitoring intra‐familial exposure to the virus [21].

**FIGURE 3:**
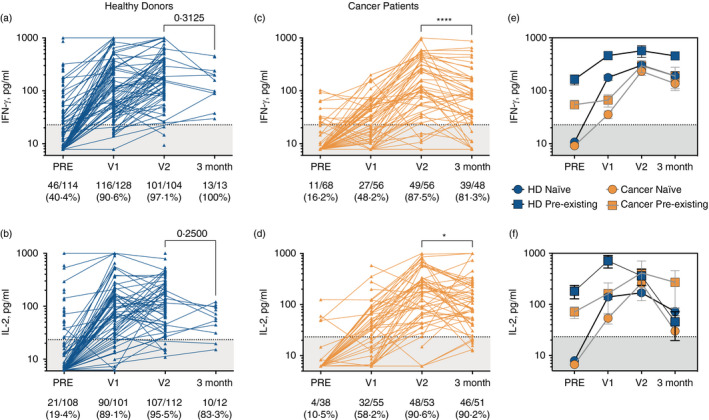
Utilizing SARS‐CoV‐2‐specific T‐cell response measurements as a read‐out for COVID‐19 vaccine efficacy amongst healthy donor and cancer patient cohorts. SARS‐CoV‐2‐specific T‐cell responses were measured using the whole‐blood assay at indicated time‐points immediately before (‘PRE’), 3–6 weeks after first dose of COVID‐19 vaccination (‘V1’), 3–6 weeks after second dose (‘V2’) and 3 months after second dose in healthy donors (A; IFN‐γ^+^, B; IL‐2^+^) and cancer patients (C; IFN‐γ^+^, D; IL‐2^+^). Two‐tailed P values resulting from Wilcoxon matched‐pairs signed rank tests are shown (* *p* < 0·05; **** *p* < 0·0001). Accumulated responses amongst healthy donors and cancer patients were monitored over the course of the vaccination schedule and separated based on pre‐existing IFN‐γ^+^ or IL‐2^+^ T‐cell responses being present prior to vaccination (squares indicate pre‐existing response, circles indicate no pre‐existing response/‘naïve’) (E and F, respectively)

Finally, the whole‐blood assay was used to track T‐cell responses immediately prior and 3–6 weeks following each SARS‐CoV‐2 vaccination dose amongst a cohort of healthy controls and a cohort of cancer patients with solid tumours. Robust priming of SARS‐CoV‐2‐specific T cells was observed amongst healthy controls, with 116 of 128 (90·6%) mounting an IFN‐γ^+^ response >22·70 pg/ml (Figure 3A) and 90 of 101 (89·1%) mounting IL‐2^+^ responses >23·31 pg/ml (Figure 3B) following a single dose. In contrast, there was a highly significant reduction in the proportion of cancer patients mounting T‐cell responses following the first dose (*p* < 0·0001, Table 2), whereby only 27 of 56 (48·2%) and 32 of 55 (58·2%) cancer patients mounted an IFN‐γ (Figure 3C) or IL‐2 (Figure 3D) response, respectively. Whilst the vaccination‐induced T‐cell response in cancer patients recovered to similar levels observed for healthy donors following the second dose (Figure 3E,F), a significant reduction in the overall proportion of cancer patients mounting IFN‐γ^+^ (*p* = 0·034) but not IL‐2^+^ (*p* = 0·29) SARS‐CoV‐2‐specific T‐cell responses remained (Table 2). In addition, healthy donors with a pre‐existing SARS‐CoV‐2‐specific T‐cell response (as a result of prior infection or perhaps cross‐reactivity) only required one dose of vaccine to induce IFN‐γ and IL‐2 responses with a greater magnitude greater than naïve (no pre‐existing T‐cell response) donors receiving two doses (Figure 3E,F).

**TABLE 2:** Comparison of vaccine‐induced SARS‐CoV‐2‐specific T‐cell responses generated by healthy donors and cancer patients

Time‐point	Cohort	IFN‐γ			IL−2		
		NR	R	*p* value	NR	R	*p* value
PRE	HD	68	46	*** *p* = 0·0009	87	21	NS *p* = 0·23
	Cancer	57	11		34	4	
V1	HD	12	116	**** *p* < 0·0001	11	90	**** *p* < 0·0001
	Cancer	29	27		33	32	
V2	HD	3	101	* *p* = 0·034	5	107	NS *p* = 0·29
	Cancer	7	49		5	48	
3 month	HD	0	13	NS *p* = 0·18	2	10	NS *p* = 0·61
	Cancer	9	39		5	46	

Note

Vaccination response rates (NR = no response, R = response) in terms of positive T‐cell (IFN‐γ, IL‐2) response amongst each cohort are shown. p values indicate results from Fisher's exact test.

*p < 0.05, **p < 0.01, ***p < 0.001, ****p < 0.0001.

Although our healthy donor cohort numbers are limited at 3 months following vaccination, T‐cell responses were generally sustained amongst this cohort. In contrast, cancer patients demonstrated a significant reduction in IFN‐γ (*p* < 0·0001; Figure 3C) and IL‐2 (*p* = 0·026; Figure 3D) SARS‐CoV‐2‐specific T‐cell responses at 3 months post‐vaccination, with several patients notable for sudden, large decreases in T‐cell responses. Whether this was the result of certain treatments or tumour progression remains to be elucidated.

Furthermore, anti‐SARS‐CoV‐2 RBD IgG antibody responses were also compromised in cancer patients following a single vaccine dose, though to a lesser degree than the T‐cell response, with 43 of 54 (79·6%) cancer patients vs. 81 of 84 (96·4%) healthy controls reaching the threshold of positivity in our antibody test (Fisher's exact test, *p* = 0020; Figure S3). A decline in IgG responses was also observed at 3 months in 14 of 37 (37·8%) patients, though this did not reach significance overall (*p* = 0·09; Figure S2B). These data may reflect a limitation of the whole blood‐based SARS‐CoV‐2 T‐cell assay, in that insufficient numbers of T lymphocytes may be present to detect such a response, a problem likely exacerbated in cancer given that lymphopenia is a common occurrence.

Overall, these data highlight the power of measuring SARS‐CoV‐2‐specific T‐cell responses as a means for identifying prior COVID‐19 infection, vaccination efficacy and/or potential future immunity from reinfection status.

## DISCUSSION

This study demonstrates the utility of a high‐throughput, standardizable T‐cell immunoassay to accurately detect SARS‐CoV‐2‐specific T‐cell responses. In order to control future outbreaks and identify at‐risk individuals, the exact constituents of effective COVID‐19 immunity at a population level must be understood. When used alongside measurements of virus‐specific antibodies, T‐cell response read‐outs represent a powerful, additional measure of potential immunity from COVID‐19, with a higher degree of confidence than either measurement on their own, in particular given the concern on the longevity of measurable antibody responses [22, 23, 24]. In addition, the FDA’s decision to issue emergency use authorization for a SARS‐CoV‐2 T‐cell test highlights the growing acceptance and usefulness of T‐cell testing for the clinical management of certain patient groups [25].

Here, we show that measuring plasma T_H_1‐type effector cytokines from SARS‐CoV‐2 peptide‐stimulated whole blood can accurately detect the presence of a cellular immune response to SARS‐CoV‐2, distinguishing those who have received prior vaccination and/or infection from uninfected healthy donors with a high degree of sensitivity and specificity. These results are consistent with comprehensive analyses of the cytokine profile released by SARS‐CoV‐2‐specific T cells measured in whole blood‐based assays or ELISpot/cell culture supernatants, which showed that IL‐2 and IFN‐γ are the dominant cytokines [5, 10, 26]. However, the relevance of an IFN‐γ‐positive, IL‐2‐negative SARS‐CoV‐2‐specific T‐cell response in uninfected donors with respect to long‐term immunity requires further investigation.

The peptides used in our immunoassay predominantly stimulate CD4^+^ T‐cell responses, and cover all major immunodominant regions of the virus, including those in the spike, membrane and nucleocapsid proteins, as recently defined [27]. T_H_1‐type responses to these immunogenic regions were detected in the vast majority of convalescent SARS‐CoV‐2‐infected and/or SARS‐CoV‐2‐vaccinated individuals, in keeping with prior findings [27, 28], although convalescent asymptomatic donors and a minority of naïve individuals demonstrate low‐level reactivity. Prior studies have revealed the functionality and magnitude of adaptive immune responses to SARS‐CoV‐2 were significantly lower in mild cases of COVID‐19 in comparison with severe cases [5], potentially the result of lower viral loads [28, 29]. In accordance with this, our study revealed that production of IFN‐γ and IL‐2 was lower in COVID‐19‐convalescent asymptomatic participants. However, a recent longitudinal analysis of asymptomatic SARS‐CoV‐2 infection identified an increased, highly functional IFN‐γ and IL‐2 response within 3 months of infection that declined faster than in symptomatic individuals [10]. Differences in post‐infection sampling times likely account for the discrepancy in associations between SARS‐CoV‐2‐specific T‐cell responses and COVID‐19 symptom severity, given that the majority of our participants were infected ~3–6 months prior to blood sampling. However, it is encouraging to note that T‐cell responses were still present and functional over this time frame in nearly all SARS‐CoV‐2‐infected convalescent and/or SARS‐CoV‐2‐vaccinated participants, even in those up to 12 months post‐infection, corresponding well with prior studies describing the longevity of anti‐SARS‐CoV‐2 T‐cell immunity [5]. Whether these responses provide immune protection, especially to new emerging mutant variants of the SARS‐CoV‐2 virus, warrants further investigation in larger prospective cohorts. In addition, further downstream analyses incorporating other coronaviruses and additional antigenic regions are necessary to ascertain whether cross‐reactive T‐cell responses play a role in SARS‐CoV‐2 immunity.

Future studies are ongoing to evaluate the durability of these measured T‐cell responses in our participants, comparing healthy subjects with immunocompromised patients, such as those with cancer. After the first dose of vaccine in cancer patients, a significantly weaker induction of cellular and, to a lesser extent, humoral responses was found, corroborating similar observations in other studies [15], although the second dose was sufficient to boost these responses to levels seen amongst healthy donors. However, many patients exhibited poor durability of vaccination‐induced SARS‐CoV‐2‐specific T‐cell responses, reducing to negative levels by 3 months post‐vaccination. These data provide further support to recent calls for cancer patients to be closely monitored for longer‐term immunological monitoring and prioritized for booster vaccines.

In summary, we describe an immunoassay that accurately and rapidly identifies the presence of SARS‐CoV‐2‐specific T‐cell responses, both helping to elucidate the adaptive immune status of previously infected and/or vaccinated individuals, and diagnosing previously unsuspected past infection. Incorporating qualitative T‐cell response data in population immunity studies, or individualized certifications of immunity, could have far‐reaching implications for government policy on future lockdown restrictions, and more effectively assess vaccine efficacy in communities, highlighting the potential requirement for repeat vaccinations where immunity wanes.

## CONFLICT OF INTEREST

MJS is a founder of and holds equity in ImmunoServ Ltd. AnG is a founder of ImmunoServ Ltd. All other authors declare no conflicts of interest.

## AUTHOR CONTRIBUTIONS

M. Scurr, A. Gallimore and A. Godkin conceptualized and designed the study. M. Scurr, W. M. Zelek, M. Evans, L. Fairclough, P. Tighe, M. Wills, B. P. Morgan, A. Gallimore and A. Godkin developed methodology. M. Scurr, W. M. Zelek, G Lippiatt, M. Somerville, S. E. A. Burnell, L. Capitani, K. Davies, H. Lawton, T. Tozer, T. Rees, K. Roberts, A. Jackson, C. Young and A. Godkin acquired data. M. Scurr, W. M. Zelek, G Lippiatt, M. Somerville, S. E. A. Burnell, L. Capitani, B. P. Morgan, A. Gallimore and A. Godkin analysed and interpreted the data. M. Scurr, W. M. Zelek and A. Godkin wrote the manuscript. M. Scurr, W. M. Zelek, G Lippiatt, M. Somerville, S. E. A. Burnell, L. Capitani, K. Davies, H. Lawton, T. Tozer, T. Rees, K. Roberts, M. Evans, A. Jackson, C. Young, L. Fairclough, P. Tighe, M. Wills, A. D. Westwell, B. P. Morgan, A. Gallimore and A. Godkin reviewed an revised the manuscript. G. Lippiatt, M. Somerville, S. E. A. Burnell, L. Capitani, K. Davies, H. Lawton, T. Tozer, T. Rees, K. Roberts, M. Evans, A. Jackson, C. Young, L. Fairclough, P. Tighe, M. Wills and A. D. Westwell provided administrative, technical or material support.

## CONSENT TO PUBLISH

All authors consent to the material to publish.

## Supporting information

Fig S1‐S3
