# Supplementary material for: Whole blood‐based measurement of SARS‐CoV‐2‐specific T cells reveals asymptomatic infection and vaccine immunogenicity in healthy subjects and patients with solid‐organ cancers
Source: Immunology. 2021 Dec 6;165(2):250–9. doi: 10.1111/imm.13433 (PMC8653009; doi:10.1111/imm.13433)
Supplement: Supplementary file 1 — Fig S1‐S3 [file IMM-165-250-s001.pdf]

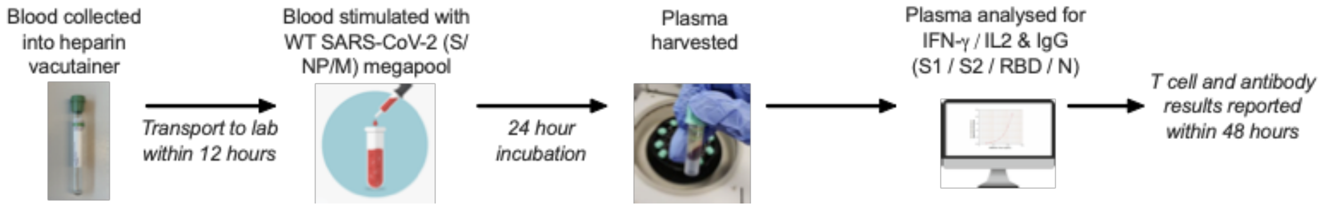

Supplementary Figure 1. Overview of the SARS-CoV-2 T cell whole blood assay.

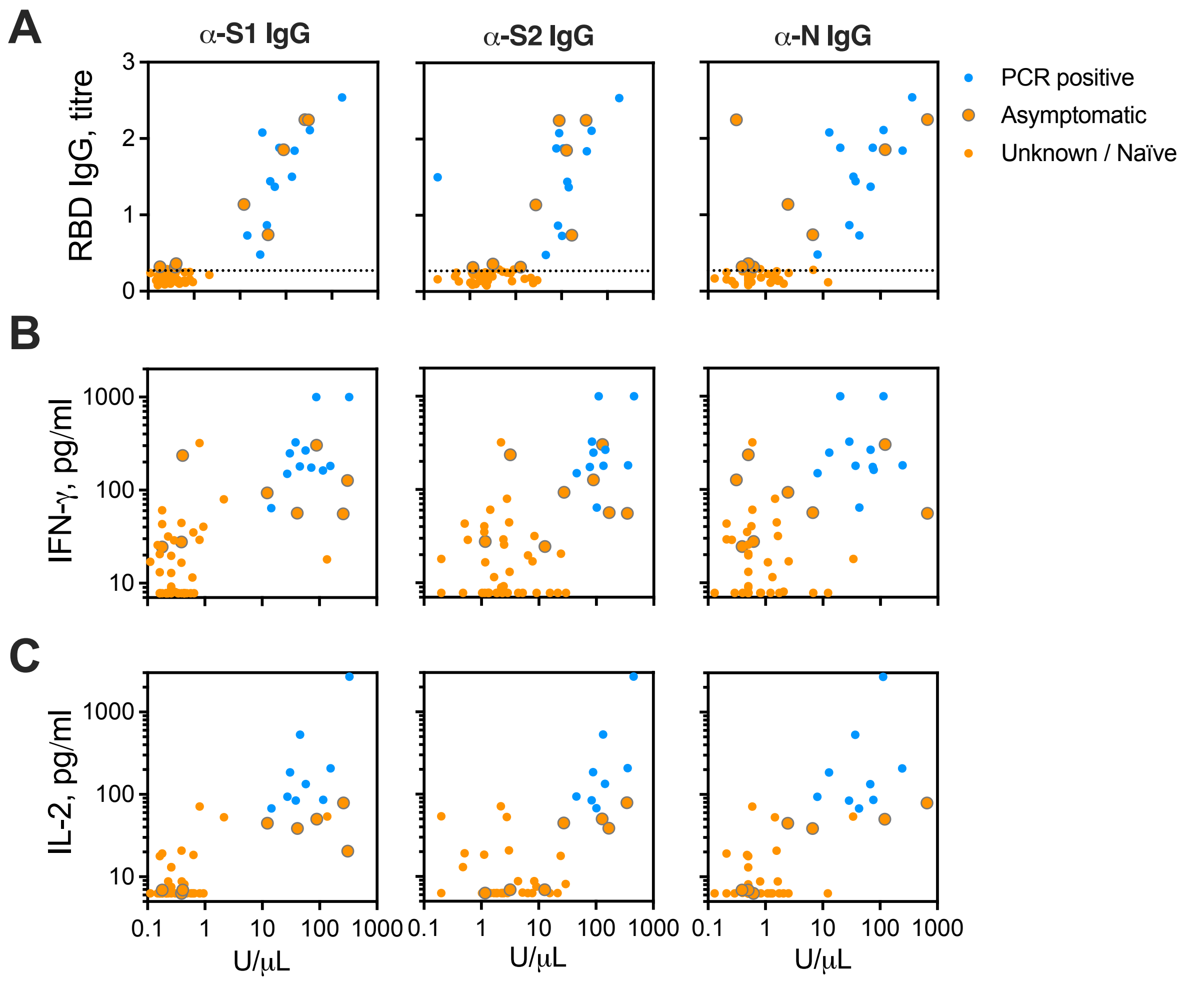

Supplementary Figure 2. SARS-CoV-2 cellular and humoral immune responses induced by prior infection. Anti-SARS-CoV-2 S1, S2 and N IgG antibodies were correlated with SARS-CoV-2 RBD IgG response (A), IFN-γ^+ T cell response (B) and IL-2^+ T cell response (C) subdivided by participant infection status, as indicated.

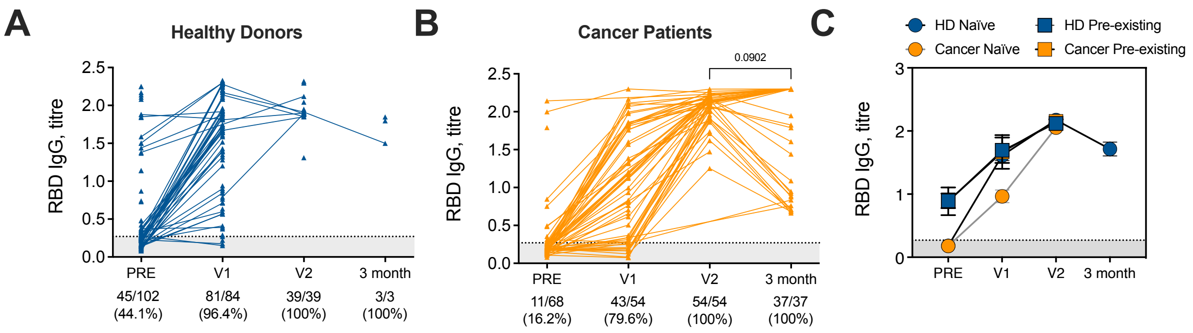

Supplementary Figure 3. Anti-SARS-CoV-2 RBD IgG response measurements as a readout for COVID-19 vaccine efficacy amongst healthy donor and cancer patient cohorts. SARS-CoV-2 RBD IgG responses were measured by ELISA at indicated time points immediately before ('PRE'), 3-6 weeks after first dose of COVID-19 vaccination ('V1'), 3-6 weeks after second dose ('V2') and 3 months after second dose in healthy donors (A) and cancer patients (B). Accumulated responses amongst healthy donors and cancer patients were monitored over the course of the vaccination schedule and separated based on pre-existing anti-RBD IgG responses being present prior to vaccination (squares indicate pre-existing response, circles indicate no pre-existing response / 'naïve') (C).
